# A Novel Regimen for Treating Melanoma: MCL1 Inhibitors and Azacitidine

**DOI:** 10.3390/ph14080749

**Published:** 2021-07-30

**Authors:** Chiara R. Dart, Nabanita Mukherjee, Carol M. Amato, Anabel Goulding, Morgan MacBeth, Robert Van Gulick, Kasey L. Couts, James R. Lambert, David A. Norris, William A. Robinson, Yiqun G. Shellman

**Affiliations:** 1 Department of Dermatology, School of Medicine, University of Colorado Anschutz Medical Campus, Mail Stop 8127, Aurora, CO 80045, USA; chda5265@colorado.edu (C.R.D.); nabanita.mukherjee@cuanschutz.edu (N.M.); david.norris@cuanschutz.edu (D.A.N.); 2 Division of Medical Oncology, School of Medicine, University of Colorado Anschutz Medical Campus, Mail Stop 8117, Aurora, CO 80045, USA; carol.amato@cuanschutz.edu (C.M.A.); morgan.macbeth@cuanschutz.edu (M.M.); robert.vangulick@cuanschutz.edu (R.V.G.); kasey.couts@cuanschutz.edu (K.L.C.); william.robinson@cuanschutz.edu (W.A.R.); 3 College of Osteopathic Medicine, Western University of Health Sciences, Pomona, CA 91766, USA; anabel.goulding@westernu.edu; 4 Department of Pathology, School of Medicine, University of Colorado Anschutz Medical Campus, Mail Stop 8104, Aurora, CO 80045, USA; jim.lambert@cuanschutz.edu; 5 Department of Veterans Affairs Medical Center, Dermatology Section, Denver, CO 80220, USA; 6 Gates Center for Regenerative Medicine, University of Colorado Anschutz Medical Campus, Aurora, CO 80045, USA

**Keywords:** melanoma, azacitidine, BH3 mimetic

## Abstract

Although treatment options for melanoma patients have expanded in recent years with the approval of immunotherapy and targeted therapy, there is still an unmet need for new treatment options for patients that are ineligible for, or resistant to these therapies. BH3 mimetics, drugs that mimic the activity of pro-apoptotic BCL2 family proteins, have recently achieved remarkable success in the clinical setting. The combination of BH3 mimetic ABT-199 (venetoclax) plus azacitidine has shown substantial benefit in treating acute myelogenous leukemia. We evaluated the efficacy of various combinations of BH3 mimetic + azacitidine in fourteen human melanoma cell lines from cutaneous, mucosal, acral and uveal subtypes. Using a combination of cell viability assay, BCL2 family knockdown cell lines, live cell imaging, and sphere formation assay, we found that combining inhibition of MCL1, an anti-apoptotic BCL2 protein, with azacitidine had substantial pro-apoptotic effects in multiple melanoma cell lines. Specifically, this combination reduced cell viability, proliferation, sphere formation, and induced apoptosis. In addition, this combination is highly effective at reducing cell viability in rare mucosal and uveal subtypes. Overall, our data suggest this combination as a promising therapeutic option for some patients with melanoma and should be further explored in clinical trials.

## 1. Introduction

Melanoma is an aggressive form of skin cancer that is responsible for the majority of skin cancer related deaths [1,2]. In the past decade, the approval of targeted therapeutics such as BRAF and MEK inhibitors and immunotherapy have greatly expanded the treatment options for melanoma patients [3,4]. However, due to primary or acquired resistance, or lack of molecular targets, these treatments are not a viable option for all patients. In particular, there is an unmet need for novel treatment options for patients with rare mucosal, uveal or acral subtypes, which infrequently harbor a BRAF mutation and generally have a low response rate to immunotherapy [5,6,7,8,9,10,11].

Cancer cell survival is achieved in part by avoiding apoptosis, a process that is regulated by the BCL2 family of proteins. This family includes both pro- and anti-apoptotic members, and their interactions among each other can control cell fate. BH3 mimetics, drugs that mimic the action of pro-apoptotic members of the BCL2 family, can trigger apoptosis through selective binding to specific anti-apoptotic BCL2 family proteins. The BH3 mimetic venetoclax (ABT-199) was the first such agent to be approved by the FDA for treating advanced hematological malignancies [12]. BH3 mimetics targeting MCL1, and the pan-BCL2 inhibitor, navitoclax, have recently entered clinical trials. The combination of ABT-199 plus the hypomethylating agent azacitidine (AZA) was recently approved for the treatment of acute myeloid leukemia (AML) and is highly effective compared to the standard-of-care single agent AZA therapy [13]. This combination was also found to target leukemia stem cells and lead to a durable remission in the majority of patients [14,15,16].

The success of combination treatment of AZA plus ABT-199 in AML patients prompted us to examine the effectiveness of similar combination treatments in melanoma. In melanoma, the apoptotic machinery is dependent in part on MCL1 [17]. We assessed the effects of MCL1 inhibitors (MCL1i; S63845, S64315) in combination with AZA in cutaneous and rare melanoma subtypes. Our data demonstrate that the combination of MCL1i + AZA can induce apoptosis, reduce proliferation, and target melanoma initiating cells (MICs) in vitro. Importantly, we found that this combination was particularly effective in rare subtypes of melanomas that lack effective treatment options, including mucosal and uveal melanoma. In this report, we demonstrate that combined treatment of MCL1i and AZA may be a potential efficacious therapeutic option for both cutaneous and rare melanoma patients.

## 2. Results

### 2.1. Single Agent or Combination of ABT-199 Plus Azacitidine Is Not Highly Effective at Reducing Melanoma Cell Viability

**Figure 1:**
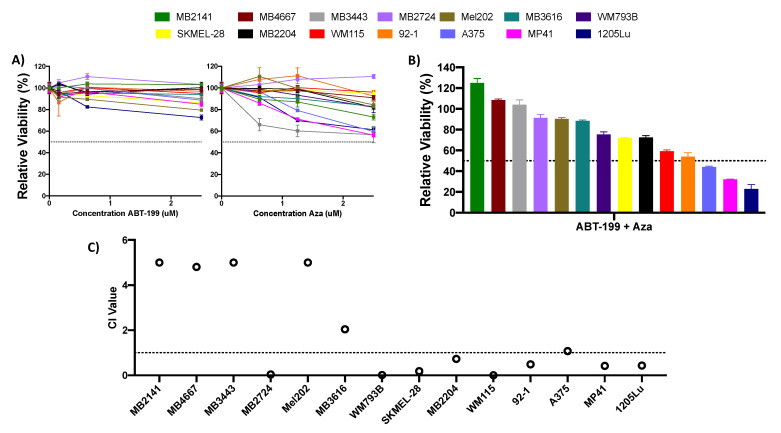
Single agent or combination treatment with ABT-199 and azacitidine (AZA) is ineffective in melanoma cell lines. (**A**,**B**) ATP assay of melanoma cell lines after 48 h of treatment with single agent AZA or ABT-199 (**A**), or their combination (**B**). Combination treatment was treated at 2.5 uM. Black dotted line indicates viability of 50%. Y axis indicates viability relative to DMSO control, set to 100%. X axis indicates drug treatment. Error bars represent +/− SEM. (**C**) Plot of the combination index (CI) values for the ABT-199 + AZA combination at 2.5 uM concentration. CI values were calculated using the CompuSyn software (version 1). CI values >1 indicate antagonism, values 0.9–1 indicate an additive effect, and values <0.9 indicate synergy. For clarity, values >5 are cutoff at 5. Black dotted line indicates CI value of 1. Y axis indicates CI value, X axis indicates cell line.

The finding that a combination of AZA + ABT-199 had a significant benefit treating AML led us to evaluate this combination in a panel of human melanoma cell lines (Figure 1). These cell lines included samples from cutaneous, rare uveal, acral, and mucosal melanoma subtypes, including both BRAF mutant and wildtype lines (Table 1 and Table 2).

We initially examined the effect of single-agent treatment with either ABT-199 (0.156–2.5 uM) or AZA (0.625–2.5 uM). Neither of these drugs alone had a substantial effect, with no cell line reaching viability below 50% at any dose (Figure 1A). We next evaluated whether the combination of ABT-199 + AZA could improve the efficacy of either drug over that of single agents. Although the combination treatment had improved efficacy over single agents in select cell lines, this was not universal. At the highest concentration tested of 2.5 uM each of ABT-199 and AZA, only 3 (~20%) cell lines achieved viability of less than 50% after treatment (Figure 1B). Additionally, only 8 cell lines experienced a synergistic effect with the combination of ABT-199 + AZA, as determined by their combination index (CI) values (CI < 0.9) (Figure 1C). The remaining cell lines experienced an antagonistic effect after combination treatment.

### 2.2. Knockdown of BCLXL or MCL1 Sensitizes Cells to Treatment with Azacitidine

**Figure 2:**
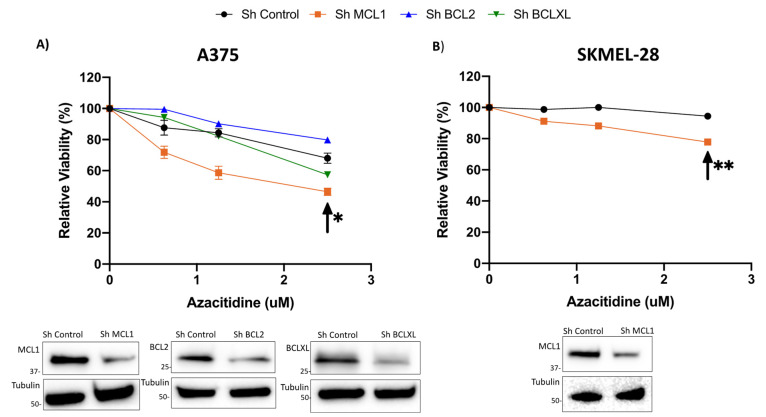
Knockdown of MCL1 or BCLXL sensitizes cells to single agent AZA treatment; knockdown of BCL2 has no effect on AZA sensitivity. (**A**) Knockdown A375 cell lines of BCL2, BCLXL, and MCL1, (**B**) Knockdown SKMEL-28 cell line of MCL1. (**A**,**B**) Knockdown cell lines were treated with AZA 0 uM- 2.5 uM for 48 h. Knockdown of MCL1 significantly sensitized cells to treatment with single agent AZA. Arrow indicates significance compared to sh Control. * indicates *p* < 0.05, ** indicates *p* < 0.01. Y axis indicates viability relative to DMSO control, set to 100%. X axis indicates AZA dosage. Immunoblot with control and knockdown cell lysates indicate knockdown of the target proteins. Error bars represent +/− SEM.

Our group and others have demonstrated that BCLXL and MCL1 are significant anti-apoptotic proteins responsible for melanoma cell survival [17,18,19]. This led us to hypothesize that inhibition of family members other than BCL2, in combination with AZA treatment, may be more potent in melanoma. To assess this, we treated BCL2, MCL1, and BCLXL knockdown cell lines with 0.625–2.5 uM AZA (Figure 2). We found that knockdown of MCL1, and to a lesser extent BCLXL, sensitized cells to treatment with AZA up to a dose of 2.5 uM, indicating that a combination of MCL1 or BCLXL inhibition with AZA treatment may be an effective combination in melanoma (Figure 2A,B). This result was particularly noteworthy for the MCL1 knockdown cell lines (A375 *p* < 0.05; SKMEL-28 *p* < 0.01) (Figure 2A,B).

### 2.3. MCL1i, S63845 or Clinical Grade Version S64315/MIK665, in Combination with Azacitidine Effectively Reduces Cell Growth in Melanoma Cells from a Variety of Subtypes

**Figure 3:**
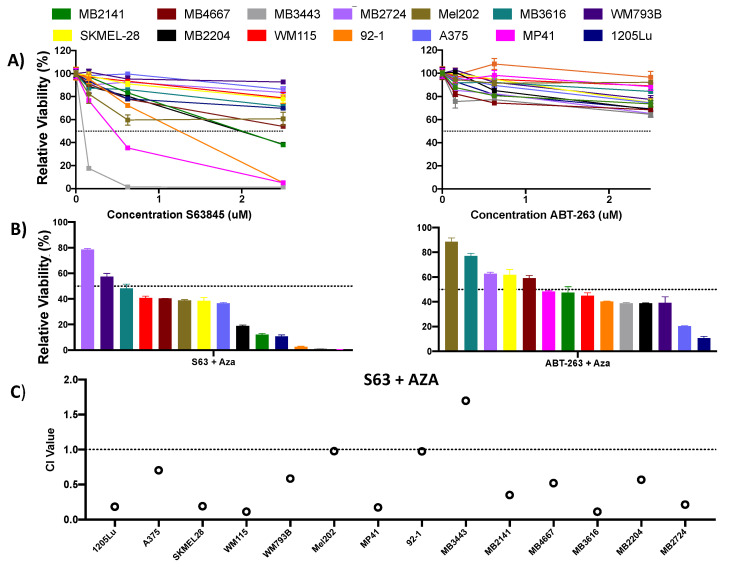
Combination treatment with S63845 + AZA is effective in synergistically reducing melanoma cell viability. (**A**) Single agent S63845 and ABT-263 dosed at a concentrations of 0.156–2.5 uM in human melanoma cell lines. (**B**) Combination treatment with S63845 + AZA, and ABT-263 + AZA dosed at 2.5 uM each in human melanoma cell lines. (**A**,**B**) All cells were treated for 48 h. Dotted line indicates 50% viability. Y axis indicates viability relative to DMSO control, set to 100%. X axis indicates drug treatment. Error bars represent +/− SEM. (**C**) CI values for S63845 + AZA treatment. CI <0.9 indicates synergy, 0.9–1 indicates additivity, >1 indicates antagonism. CI values are calculated at the 2.5 uM dose using CompuSyn (version 1) software. Black dotted line indicates CI value of 1. Y axis indicates CI value, X axis indicates cell line and treatment.

The results of our knockdown studies led us to evaluate the efficacy of MCL1 or BCLXL inhibitors alone and in combination with AZA (Figure 3, Figure S1). As single agents, the BH3 mimetics S63845 (MCL1i), A1331852 (BCLXL inhibitor), and ABT-263 (inhibitor of BCL2, BCLXL, and BCLW) were not universally effective in suppressing cell growth in our melanoma cell line panel (Figure 3A, Figure S1A). Although some cell lines were sensitive to the MCL1i S63845 (Figure 3A), we found that all three combinations had improved efficacy compared to the combination of ABT-199 and AZA (Figure 3B, Figure S1B). Of these, the most promising was the combination of the MCLi S63845 + AZA, with ~85% of cell lines responding with a viability of <50% at 2.5 uM of S63845 and AZA. Additionally, the combination treatment at 2.5 uM of each drug had a synergistic effect in 11 cell lines, the remaining cell lines experienced a synergistic effect at lower doses (Figure 3C, Figure S2). 2 cell lines experienced an additive effect at the 2.5 uM concentration.

**Figure 4:**
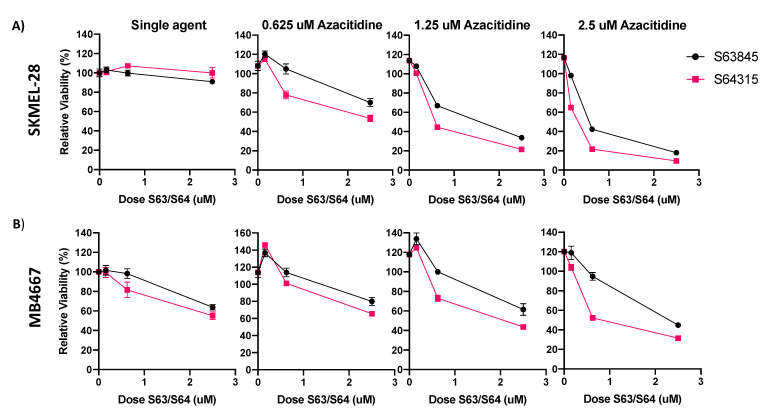
S63845 and clinical grade version S64315 (MIK665) have similar efficacy in melanoma cell lines. (**A**) ATP assay of S63845 or S64315 plus AZA in SKMEL-28. (**B**) ATP assay of S63845 or S64315 plus AZA in MB4667. Dose of AZA indicated in title of graph. Y axis indicates viability relative to DMSO control, set to 100%. X axis indicates S63845/S64315 dosage. Error bars represent +/− SEM.

We thus focused on the combination of MCL1i + AZA for the remainder of our studies. We also evaluated the efficacy of S64315 (MIK665), the clinical grade counterpart of S63845 (Figure 4). We compared the activity of the S64315 to S63845 as single drug and in combination with AZA in two cell lines with average sensitivity to S63845 + AZA treatment (Figure 4A,B). Overall, S64315 performed similarly, and in some cases slightly more effective than S63845, either alone or in combination with AZA. S64315 + AZA was synergistic in both cell lines evaluated (data not shown).

### 2.4. MCL1i Plus Azacitidine Induce Apoptosis and Reduce Proliferation in Melanoma Cell Lines

**Figure 5:**
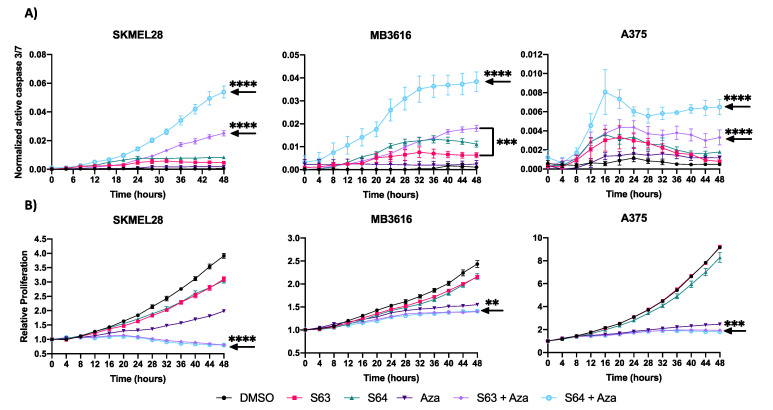
Treatment with S63845/S64315 plus AZA decreases proliferation and induces apoptosis. (**A**) IncuCyte live cell analysis of caspase 3/7 activity. Y axis indicates ratio of area expressing fluorescent signal. X axis indicates time in hours. (**B**) IncuCyte live cell analysis of cellular proliferation. Y axis represents confluence relative to 0 h. X axis indicates time in hours. For all graphs, error bars represent +/− SEM. Arrow indicates lowest significance compared to all other conditions. ** indicates *p* < 0.01, *** indicates *p* < 0.001 **** indicates *p* < 0.0001.

To determine how the combination of MCL1i + AZA reduced melanoma cell growth, we utilized IncuCyte live cell imaging with a fluorescent caspase 3/7 reagent to monitor apoptosis and cellular proliferation after various treatments (Figure 5, Figure S3). Cells were treated over 48 h with DMSO control, single agent of AZA or MCL1i (S63845 or S64315), or the combination of MCL1i + AZA. Overall, combination treatments with MCL1i + AZA had the highest caspase 3/7 activity in all cell lines tested, significantly higher over control or single agent treatments in all but two cell lines (MB2141, MB3616) (Figure 5A, Figure S3A). In addition, the combination treatments decreased proliferation compared with DMSO or single agents (Figure 5B, Figure S3B), statistically significant in all cell lines, except two (MB2141, MB4667). Thus, these data suggest the combination treatments were most effective to induce apoptosis and reduce proliferation.

### 2.5. MCL1i Plus Azacitidine Decreases Sphere Formation of Melanoma Cell Lines

**Figure 6:**
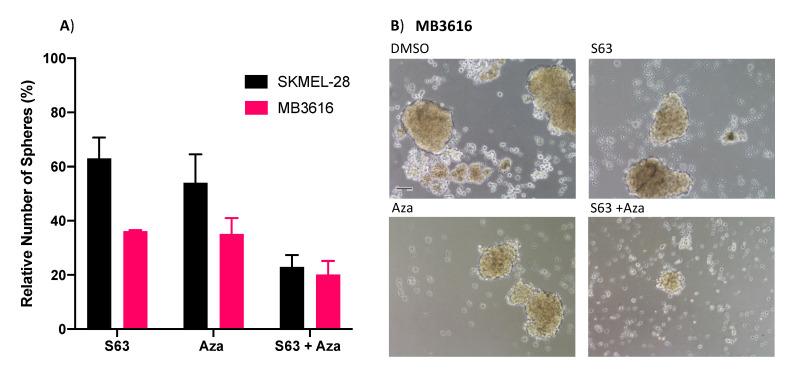
S63845 plus AZA treatment disrupts sphere formation. (**A**) Quantification of primary spheres after 48 h of indicated treatments. Y axis represents sphere number relative to DMSO control, set to 100%. X axis indicates treatment. Error bars represent +/− SEM. (**B**) Brightfield images of representative wells of MB3616 spheres after 48 h of indicated treatment. Scalebar represents 100 um.

Prior studies have shown that in melanoma there is a subset of cells termed melanoma initiating cells (MICs), which share some features with cancer stem cells (CSCs) [20,21,22]. This subset of cells has been implicated in relapse to targeted therapies, suggesting that any effective long-term treatment must target these cells as well as bulk tumor cells. The primary sphere assay measures the impact of a drug treatment on the viability of CSCs, including MICs. This technique hinges upon the ability of CSCs to survive and form spheres in nonadherent, serum-free conditions [19,23,24,25,26]. We examined the effect of combination treatment with S63845 + AZA on sphere formation using two representative melanoma cell lines, with mid-range sensitivity to combination treatment (SKMEL-28, MB3616) (Figure 6A,B). This combination significantly decreased sphere formation compared to DMSO controls (*p* < 0.001), or single agents (Figure 6A). Additionally, brightfield imaging showed substantially disrupted spheres in wells treated with combination versus single agent or control (Figure 6B).

### 2.6. Sensitivity to MCL1i Plus Azacitidine Treatment May Be Stratified by Melanoma Subtype

**Figure 7:**
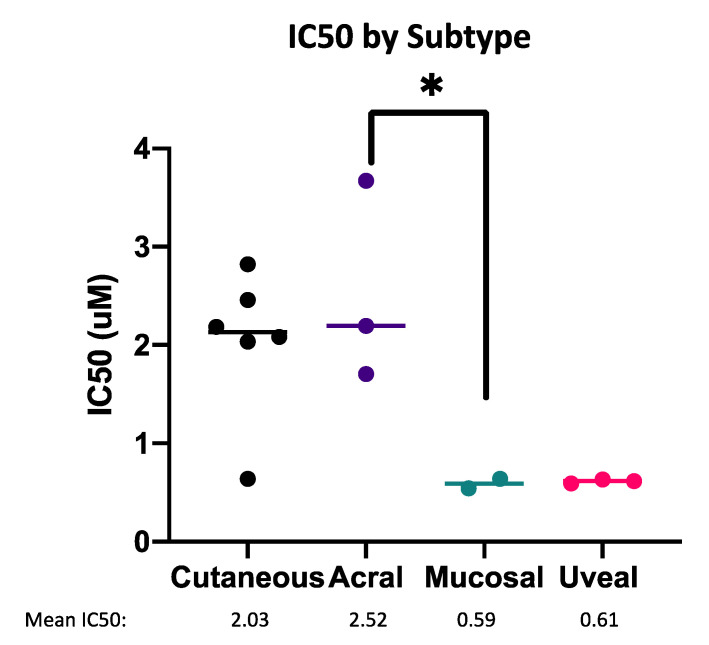
Mucosal and uveal melanoma are most sensitive to MCL1i + AZA. Dot plot of IC50 values for the combination of S63845 + AZA, separated by melanoma subtype. Each dot represents one cell line. * represents *p* < 0.05.

We investigated variables that may affect the sensitivity of melanoma cells to the combination of MCL1i + AZA (Figure 7, Figure S4). These included the status of driver mutations, melanoma subtypes, and expression of pro-apoptotic BCL2 family members. Interestingly, melanomas of rare uveal or mucosal subtypes had greater sensitivity (lower IC50) than cutaneous or acral melanomas (Figure 7). Due to limited sample size, this comparison was only significant between the acral and mucosal subtypes (*p* < 0.05).

### 2.7. Cell Death Induced by MCL1i Plus Azacitidine Is Not Dependent on BIM or NOXA

Previously, expression of pro-apoptotic BCL2 family members (BIM and NOXA) have been identified as important for killing effects of combinations with BH3 mimetics in melanoma [23]. However, knockdown of either BIM or NOXA did not show consistent alteration of sensitivity to the combination treatment of MCL1i plus AZA, indicating that these proteins may not play a major role in sensitivity to this treatment (Figure S4B).

Overall, these data suggest that a treatment combining MCL1 inhibition and AZA is most beneficial for melanoma patients from the rare mucosal and uveal subtypes, for which there are limited treatment options currently available.

## 3. Discussion

The approval of immune checkpoint blockade and targeted therapies has greatly expanded treatment options for patients with cutaneous melanoma, however, relapse or non-response to current treatments is still a major issue for a large fraction of cutaneous melanoma patients [4,27,28]. Moreover, patients with rare melanomas such as uveal or mucosal have even more restricted treatment options, for instance, uveal melanomas have only one FDA approved drug tebentafusp. However, this treatment is limited to only 50% of uveal melanoma patients, due to its reliance on one specific HLA type [29,30]. Mucosal melanoma patients face a similar scenario, with only one approved drug; nemvaleukin was recently granted orphan drug status to treat mucosal melanoma [31,32]. Both of the above approved drugs for uveal and mucosal melanomas are immunotherapy drugs and there is no known biomarker to predict which patients will respond. Thus, there is still an unmet need for new treatment options for these patients.

Activating the apoptotic pathway using BH3 mimetics is a unique way of killing cancer cells, which has been very successful in hematological cancers. BH3 mimetics antagonize the action of anti-apoptotic proteins such as BCL2, MCL1 etc. Previous studies by our group and others indicate that combination treatments targeting BCL2 family proteins, particularly MCL1, may be a promising option in melanoma [17,19,23]. The combination treatment of AZA plus ABT-199 was recently approved for the treatment of some leukemias. This combination vastly improved the treatment efficacy over the historical standard-of-care single agent AZA (complete remission + complete remission with incomplete hematologic recovery 66.4% vs. 28.3%, median overall survival 14.7 vs. 9.6 months) [14]. This treatment was also shown to target leukemia stem cells in AML and has revolutionized the treatment of AML patients not eligible for more cytotoxic chemotherapeutics [13,15]. In this study, we evaluated similar combinations of AZA plus BH3 mimetics in melanoma. We found that the clinically approved combination of ABT-199 plus AZA was not effective in our panel of melanoma cell lines. We demonstrate here that the lack of efficacy of this combination in melanoma may be due to their dependence on MCL1, rather than BCL2 for survival.

The single agent efficacy of AZA has been evaluated in uveal and cutaneous melanoma in preclinical studies and is currently being evaluated in combination with the checkpoint inhibitor pembrolizumab, or the chemotherapeutic carboplatin in phase II clinical trials (clinicaltrials.gov, NCT02816021, anzctr.org, ACTRN12618000053224). Preclinical studies with single agent AZA show that although AZA treatment was able to reduce the growth and invasiveness of bulk melanoma cells and MICs, it was incapable of inducing apoptosis in uveal melanoma cell lines, and in all but one cutaneous cell lines [33]. Our results demonstrate that the combination of AZA + MCL1i further enhances the antiproliferative effect of AZA in bulk tumor cells as well as MICs. Additionally, our results demonstrate that AZA + MCL1i can induce apoptosis in all melanoma cell lines evaluated, including uveal melanoma lines.

The studies reported here indicate that MCL1i plus AZA is the most effective combination of BH3 mimetic plus AZA in melanoma. We show that this combination can synergistically induce apoptosis and inhibit proliferation in the majority of melanoma cell lines. We also show that this combination targets MICs, a subpopulation of cells that are thought to be responsible for relapse after treatment, indicating that this treatment may be able to prevent recurrence. Notably, we show that the rare subtypes of mucosal and uveal melanoma are quite sensitive to the combination of MCL1i plus AZA. Several uveal and mucosal cell lines were sensitive to single agent MCL1i, however combination treatment was necessary to achieve both anti-proliferative and pro-apoptotic effects. Additionally, the combination treatment was synergistic in 2/5 of these cell lines at the dose of 2.5 uM each S63845 + AZA and was synergistic at a lower dose for the remaining cell lines. The treatment options for these subtypes are currently quite limited. Thus, this combination may provide a novel treatment for melanoma patients lacking therapeutic options.

Response to treatment with the combination of ABT-199 plus AZA in AML was found to be associated with mutational status of several key genes, such as NPM1, IDH1, IDH2, and DNMT3 [34]. Therefore, sensitivity to the combination of MCL1 + AZA may be similarly associated with mutational status in melanoma. Although our study did not find any significant difference in sensitivity between driver mutations, this comparison is limited by the small number of cell lines evaluated in this study. Instead, our data indicated melanomas of mucosal or uveal subtype may be most sensitive to this combination. Interestingly, the rare subtype of acral melanoma was not particularly sensitive to this combination. All rare melanoma subtypes are mostly non-UV mediated unlike cutaneous melanomas [35,36]. All of them also have their own unique genetic landscapes [37,38]. These genetic differences likely contribute to the differences in sensitivity, and it is worthy of further investigations in the future studies.

Regulation of melanocyte pigmentation overlaps both with the apoptotic pathway, as well as the commonly mutated MAPK pathway [39]. Notably, expression of the anti-apoptotic protein BCL2 is controlled in part by the pigmentation regulating protein MITF, and knockout of BCL2 in mice leads to early hair graying, indicating a potential role for pigmentation in this treatment [40,41,42]. However, of the cell lines assessed in this study, none were pigmented, and there was no obvious change in pigmentation during passaging (data not shown) or among the treatments (see an example in Figure S5). Thus, we did not have any evidence that pigmentation may play a role in this treatment.

Overall, the findings presented here suggest that MCL1 inhibition plus AZA is a promising combination in melanoma treatment. Due to the general anti-apoptotic role of MCL1, there are concerns regarding potential on-target toxicity in somatic cells [43]. However, multiple in vivo studies using MCL1i have previously been carried out in murine models with manageable toxicities [44,45]. Additionally, past studies in our group found tolerable doses of S63845 in combination with ABT-199, A1338152, or ABT-263 [19,23]. S64315 is currently being investigated in three phase I clinical trials in hematopoietic malignancies (clinicaltrials.gov, NCT02992483, NCT02979366, NCT03672695). So far, no data for the safety of these treatments have been reported.

Excitingly, there are already ongoing clinical trials evaluating two different MCL1i in combinations with AZA in AML patients, even though no in vivo data for the combination of MCL1i + AZA has been reported. One is a phase I/II clinical trial evaluating the tolerability of the combination of S64315 + AZA (clinicaltrials.gov, NCT04629443), and the other is a phase I clinical trial to evaluate another MCL1i (AMG176) + AZA (clinicaltrials.gov, NCT02675452). Our limited pilot studies indicate that the MCL1i AMG176 + AZA may have similar efficacy to S64315 + AZA (data not shown). No safety concerns for either the combination of S64315 + AZA or AMG176 + AZA have been reported to date.

So far, there is no clinical trial of MCL1i + AZA for melanoma patients. However, our data here support the idea to explore the therapeutic potential of the combination of MCL1i + AZA in certain melanoma patients. Based on the data available from leukemia studies and our in vitro data, we propose that such combinations may have significant efficacy in advanced melanoma with limited toxicity and provide a new way forward in melanoma and other cancers in the near future.

## 4. Materials and Methods

### 4.1. Cell Lines and Patient Sample Derived Lines

Patient derived cell lines were established from tumor samples of patients at the University of Colorado Hospital (study use agreement COMIRB 05-0309). Patient lines were short tandem repeat (STR) profiled, with a >80% match cutoff to peripheral blood or tumor specimen from the same patient. The cell lines MP41 and MP46 were purchased from the American Type Culture Collection (ATCC, Manassas, VA, USA), 92-1 and Mel202 were purchased from Sigma Aldrich (St. Louis, MO, USA). Cells were maintained in RPMI media with 10% FBS and 5% penicillin- streptomycin.

### 4.2. Drugs and Dosages

S63845, S64315, ABT-199, ABT-263, A1331852, and azacitidine were purchased from MedChem Express (Monmouth Junction, NJ, USA). All drugs were reconstituted as per manufacturer’s instruction using DMSO and used at a dose between 0.156 uM and 2.5 uM. Unless otherwise stated, the concentration used was 2.5 uM. For all in vitro assays, cells were treated for 48 h and kept at 36.5 °C and 5% CO_2_. Table 1 and Table 2 list the genotype and other relevant details of commercial cell lines and patient derived cell lines.

### 4.3. Cell Viability, Proliferation and Apoptosis Assays

Cells were plated at a density of 3000–5000 cells per well in tissue culture treated 96 well plates. The ATP viability assay was completed as described in our previous publication [23], using the CellTiter-Glo luminescent cell viability assay (Promega, Madison, WI, USA). The IncuCyte live cell analysis was conducted as described by our group previously [19] over 48 h at concentrations of 2.5 uM each drug unless otherwise stated. For cell pellet assay, cells were plated at a density of 400,000 cells per plate in a 10 cm dish and treated for 48 h with 2.5 uM of MCL1i + AZA.

### 4.4. Immunoblot

Lysates were collected and blots were run as described in our previous publications [46,47]. Cells were treated with 0.156–2.5 uM for 48 h in 10 cm dishes prior to collection. The antibodies used were MCL1, BCLXL, BCL2 (#819, #2764, #15071, Santa Cruz Biotechnology, Dallas, TX, USA), NOXA (#114C307, Millipore Sigma, Burlington, MA, USA) BIM, and α/β tubulin (#2933, #2148, Cell Signaling Technologies, Danvers, MA, USA). Anti-rabbit or anti- mouse IgG secondary antibodies (#7074S, #7076, Cell Signaling Technologies), were diluted 1:10,000 in 5% nonfat milk solution.

### 4.5. Creation of Stable Knockdown Cell Lines

The shRNA (short hairpin) transduced lines were created as previously described for all cutaneous melanoma lines [48].

### 4.6. Primary Sphere Forming Assay

The assay was conducted as described previously [18,49,50,51]. Briefly, cells were seeded at a density of 5k–10k cells/mL with serum-free stem cell media in non-adherent plates. After 120 h, cells were treated with DMSO, AZA, S63845, or a combination, at a concentration of 2.5 uM. After 48 h of treatment, spheres were counted and representative images were taken. Spheres were defined as cell colonies with a minimum diameter of 50 um.

### 4.7. Statistical Analysis, Calculation of IC50 and CI Values

Statistical significance was evaluated in Graphpad Prism 8 (GraphPad Software, San Diego, CA, USA) using one way ANOVA or t tests, with appropriate follow up tests. All graphs show mean +/− standard error of mean. Combination index (CI) values were calculated using CompuSyn software (version 1, (ComboSyn, Paramus, NJ, USA), using viability values from 0.156–2.5 uM single agent and combination treatment for each cell line. CI values indicate the synergistic, additive, or antagonistic effect of a drug combination. Values < 0.9 indicate synergy, values 0.9–1 indicate an additive effect, and values > 1 indicate antagonism [52]. IC50 was calculated using a variable slope (log)-response curve, as described in our previous publications [23].

## 5. Conclusions

In summary, our data indicate that the combination of MCL1 inhibitor with AZA may be a viable option for melanoma patients. This combination is particularly promising for melanomas from the rare types of uveal and mucosal, which have limited treatment options. It is noteworthy that the combination of MCL1 inhibitors with AZA is currently in clinical trials for hematological cancers, which will likely provide useful information and help in moving this combination to clinical trials for melanoma patients quickly. Therefore, our data suggest that this combination may provide a new way forward in melanoma patients lacking other treatment options.

## Figures and Tables

**Table 1:** Genotype and subtype of commercially available or patient derived cell lines used.

Cell/MB Line	Genotype	Subtype
A375	BRAF V600E	Cutaneous
1205Lu	BRAF V600E	Cutaneous
SKMEL-28	BRAF V600E	Cutaneous
WM793B	BRAF V600E	Cutaneous
WM115	BRAF V600D	Superficial spreading
MB2141	EML4-ALK	Mucosal
MB3443	NRAS Q61H	Mucosal
MB3616	NRAS Q61K	Superficial spreading
MB4667	NRAS Q61R	Acral
MB2724	Triple WT (BRAF, NRAS, NF)	Acral
MB2204	Triple WT (BRAF, NRAS, NF)	Acral
Mel202	SF3B1 R625G, GNAQ Q209L, R210K	Uveal
92-1	GNAQ Q209L	Uveal
MP46	GNAQ Q209P	Uveal
MP41	GNAQ Q209L	Uveal

**Table 2:** Details of patient derived cell lines used.

MB Line	Primary or Metastatic	Sample Collection Site	Pigmentation Status of Tumor
MB2141	Metastatic	Subcutaneous	Unknown
MB3616	Metastatic	Brain	Unknown
MB2724	Metastatic	Lymph node	Unknown
MB3443	Metastatic	Neck	Amelanotic
MB4667	Metastatic	Subcutaneous	Amelanotic
MB2204	Metastatic	Subcutaneous	Amelanotic

## Data Availability

The data presented in this study are available in the main text, Supplementary Materials, or on request from the corresponding author.

## Supplementary Materials

The following are available online at https://www.mdpi.com/article/10.3390/ph14080749/s1, Figure S1: Single agent and combination treatment with A1331852 + AZA, Figure S2: S63845 + AZA is synergistic at lower doses in select melanoma lines. Figure S3: Treatment with S63845/S64315 + AZA decreases proliferation and induces apoptosis in four cell lines. Figure S4: Driver mutation status is not related to IC50 to S63845 + AZA. Figure S5: Treatment with MCL1i + AZA does not alter pigmentation in melanoma cells.
