# Supplementary material for: A Novel Regimen for Treating Melanoma: MCL1 Inhibitors and Azacitidine
Source: Pharmaceuticals (Basel). 2021 Jul 30;14(8):749. doi: 10.3390/ph14080749 (PMC8399604; doi:10.3390/ph14080749)
Supplement: Supplementary file 1 [file pharmaceuticals-14-00749-s001.zip › Supplemental Figure Legends-MCL1i+AZA 7-19.pdf]

Supplemental Figures:

Supplemental figure S1: Combination treatment with A1331852 (BCLXL inhibitor) + AZA is effective in reducing melanoma cell viability; single agent treatment with A1331852 is ineffective in melanoma cell lines. A) Singe agent A1331852 dosed at a concentration of 0.156 – 2.5 uM in 14 human melanoma cell lines. B) Combination treatment with A1331852 + AZA at 2.5 uM each in 14 human melanoma cell lines. All cells were treated for 48h. Dotted line indicates 50% viability. Y axis indicates viability relative to DMSO control, set to 100%. X axis indicates drug treatment. Error bars represent +/- SEM.

Supplemental figure S2: S63845 + AZA is synergistic at lower doses in select melanoma lines. Plot of the CI values for the S63845 + AZA combination at 0.625 uM S63845 + 1.25 uM AZA. CI values were calculated using the CompuSyn software (version 1). CI values >1 indicate antagonism, values 0.9-1 indicate an additive effect, and values <0.9 indicate synergy. Y axis indicates CI value, X axis indicates cell line.

Supplemental figure S3: Treatment with S63845/S64315 + AZA decreases proliferation and induces apoptosis in four cell lines. A) IncuCyte live cell analysis of caspase 3/7 activity. Y axis indicates ratio of area expressing fluorescent signal. X axis indicates time in hours. B) IncuCyte live cell analysis of cellular proliferation. Y axis represents confluence relative to 0h. X axis indicates time in hours. For all graphs, error bars represent +/- SEM.

Supplemental figure S4: Driver mutation status is not related to IC50 of S63845 + AZA, knockdown of BIM or NOXA does not significantly impact treatment efficacy of AZA + S63845. A) Dot plot of IC50 values for the combination of S63845 + AZA, separated by driver mutation status. Each dot represents one cell line. B) NOXA and BIM knockdown lines were created in A375 cells using shRNA technology. Y axis indicates viability relative to DMSO control, set to 100%. X axis indicates treatment. Western blots confirm knockdown of each target protein vs. sh Control. Error bars represent +/- SEM.

Supplemental figure S5: Treatment with MCL1i + AZA does not alter pigmentation in melanoma cells. (A,B) Cell pellets after monolayer treatment for 48h with DMSO, S63845+ AZA or S64315 + AZA in MB3616 (A) or SKMEL28 (B) cell lines.
