# Supplementary material for: A Novel Regimen for Treating Melanoma: MCL1 Inhibitors and Azacitidine
Source: Pharmaceuticals (Basel). 2021 Jul 30;14(8):749. doi: 10.3390/ph14080749 (PMC8399604; doi:10.3390/ph14080749)

Supplemental figure S1

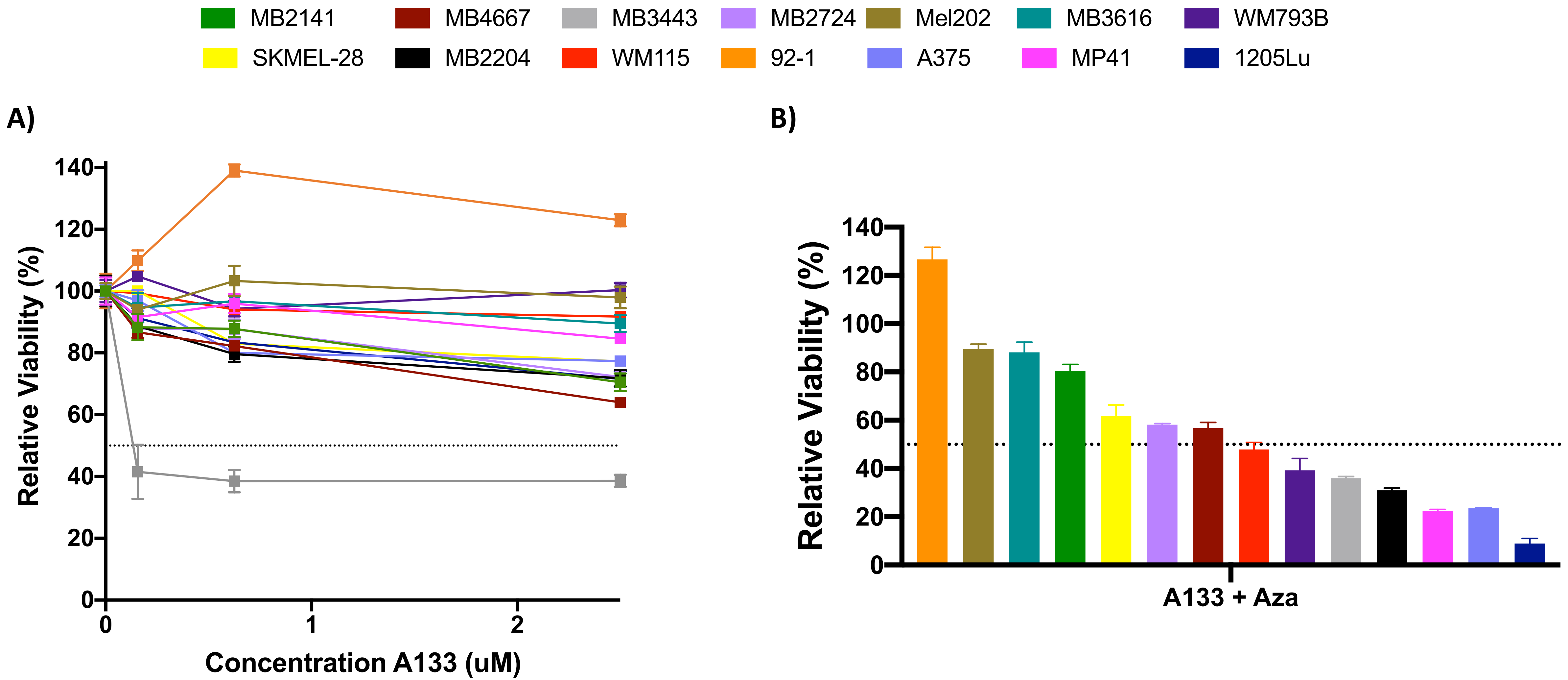

Supplemental figure S2

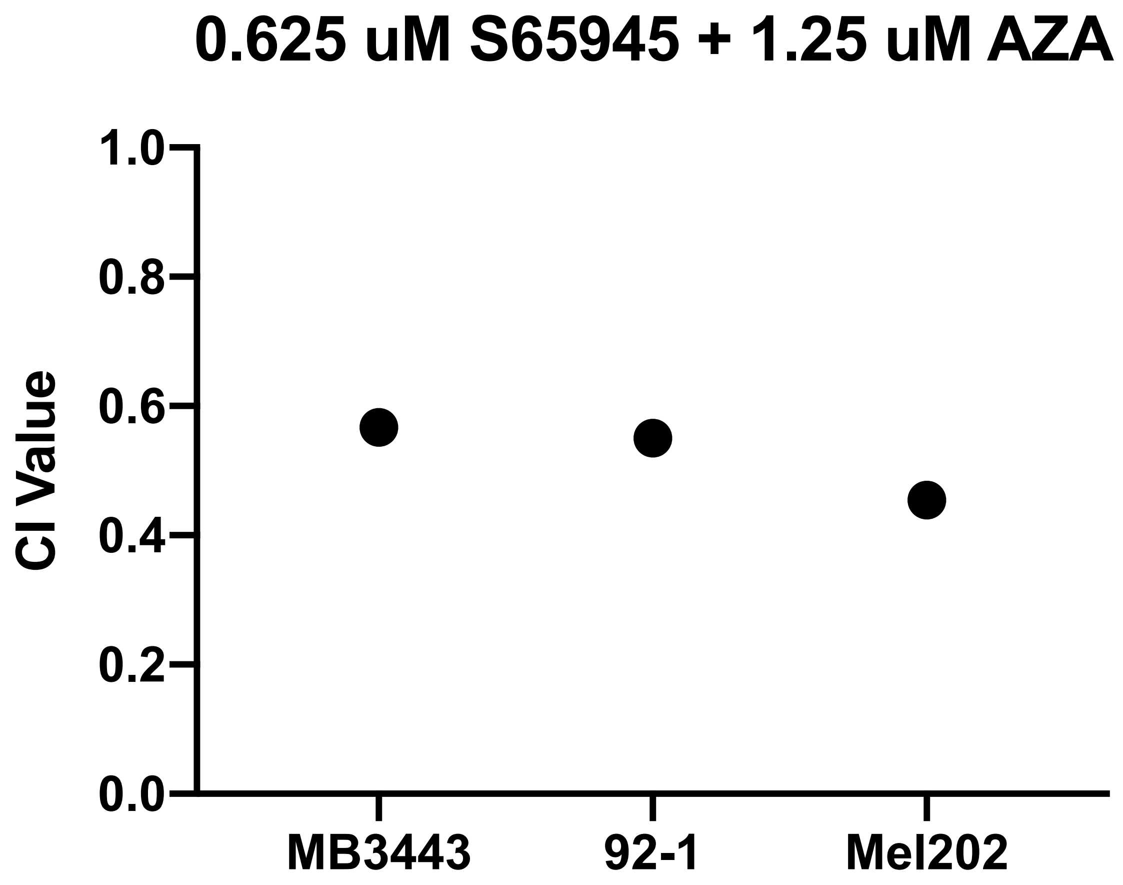

Supplemental figure S3

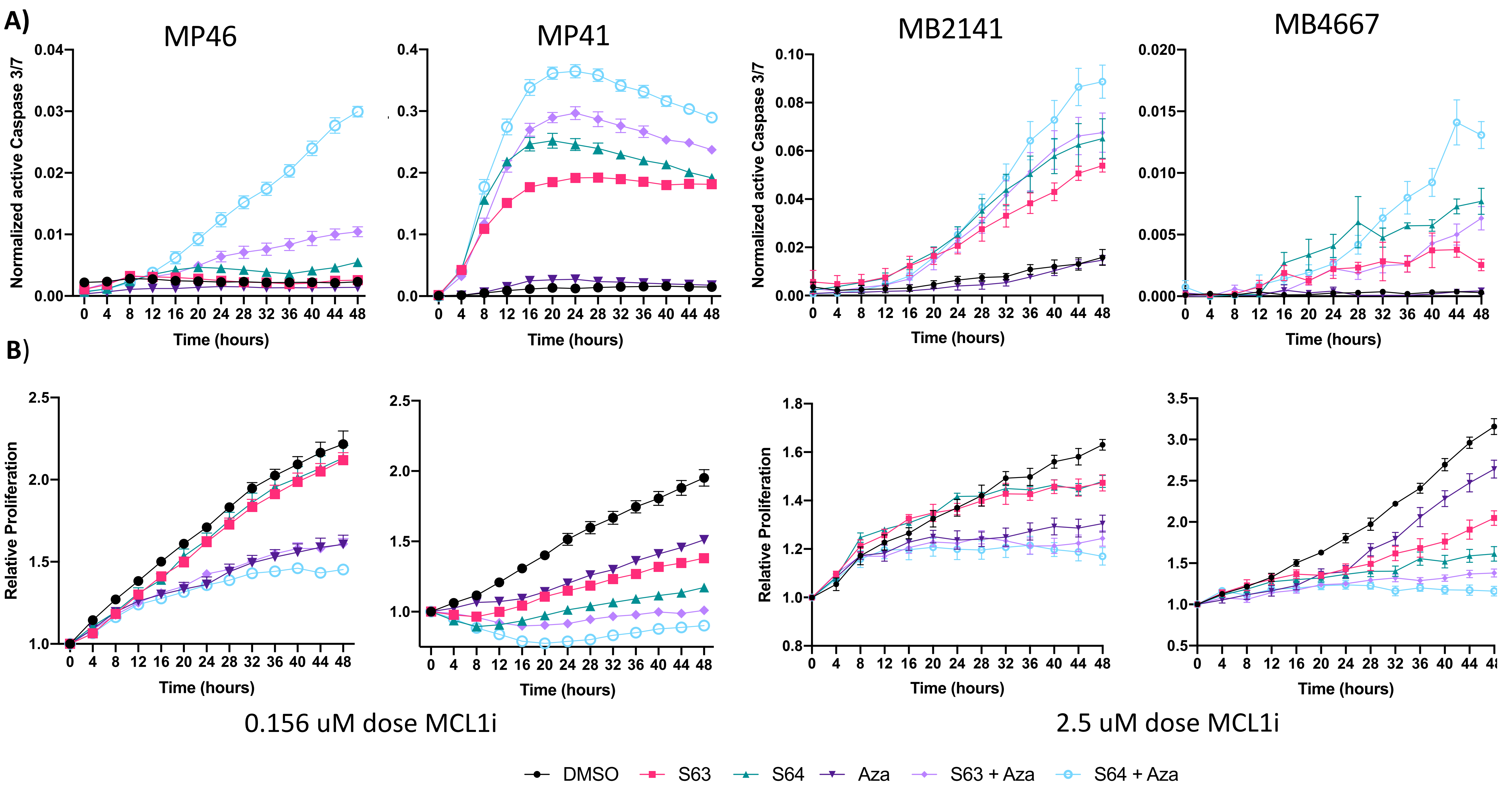

Supplemental figure S4

A)

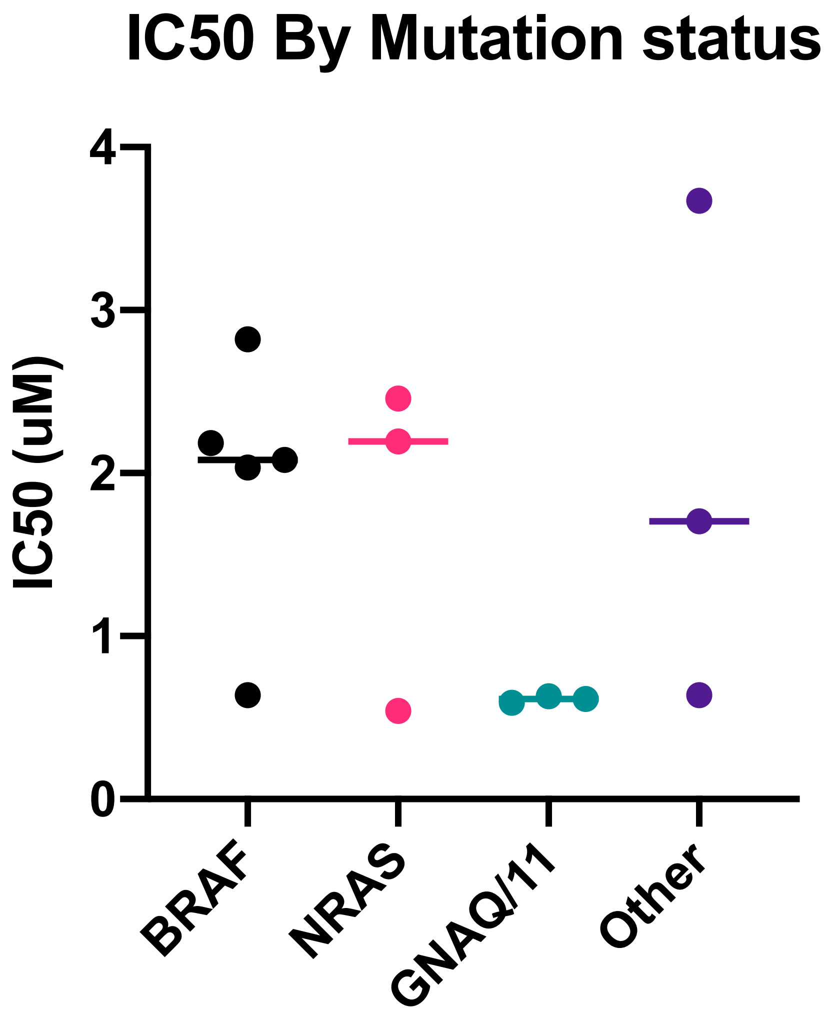

B)

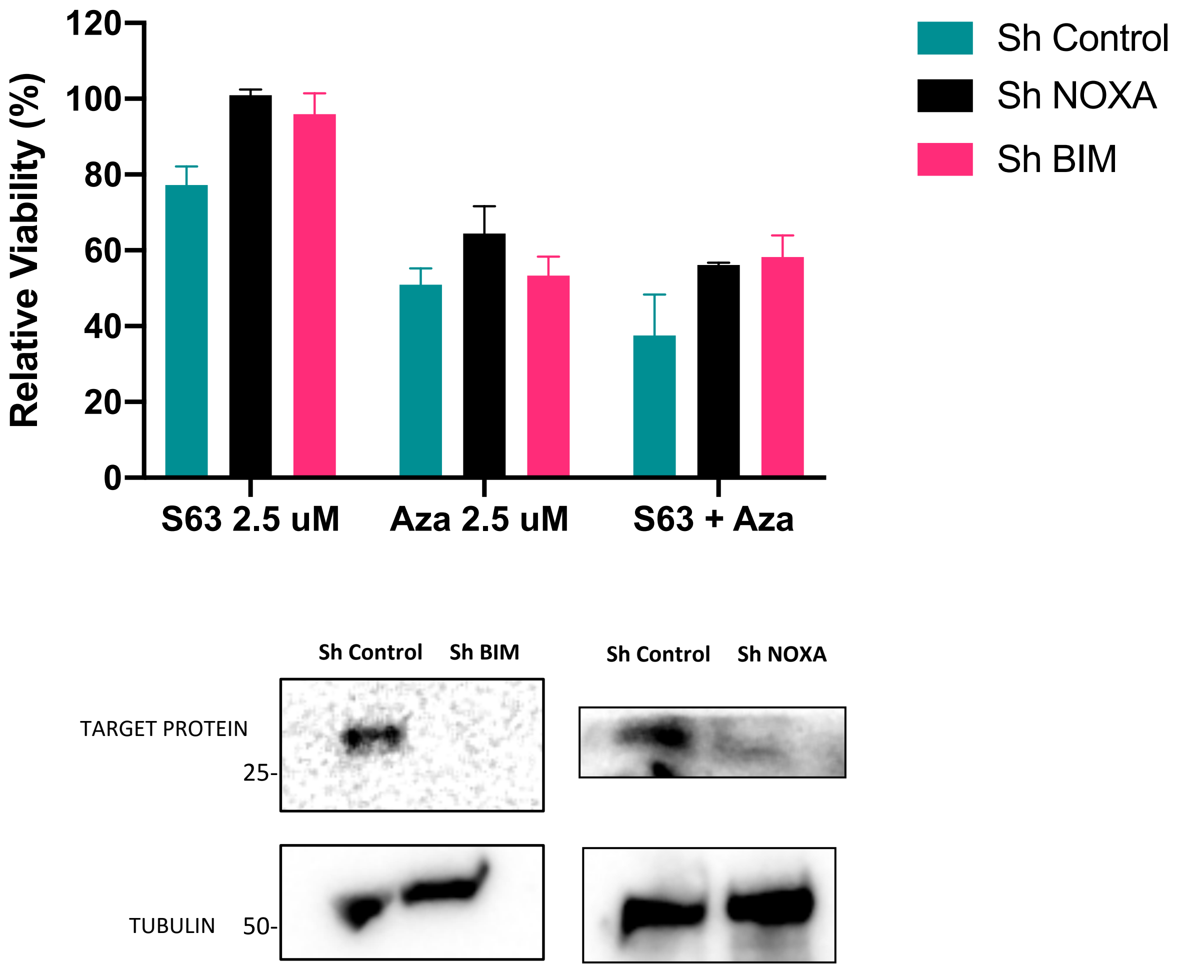

Supplemental figure S5

A) MB3616

B) SKMEL28

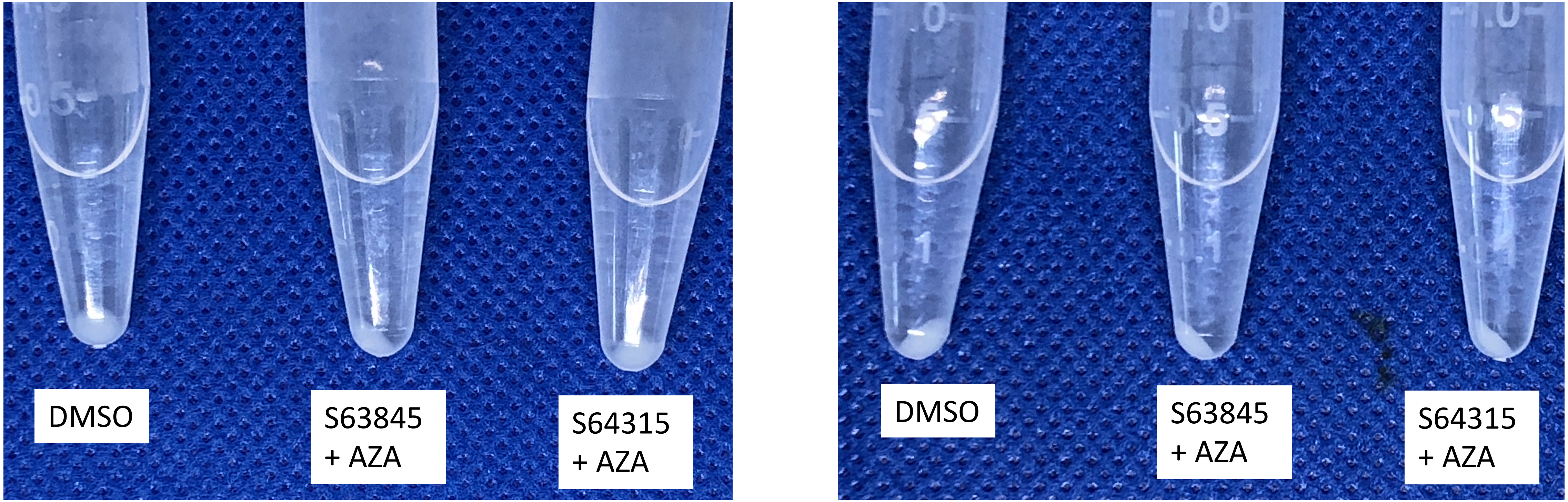

Supplement: Supplementary file 1 [file pharmaceuticals-14-00749-s001.zip › Supplemental figures-MCL1i+AZA 7-15.pdf]
